# The relationship between epicardial fat thickness and gestational diabetes mellitus

**DOI:** 10.1186/1758-5996-6-120

**Published:** 2014-11-06

**Authors:** Gökay Nar, Sinan Inci, Gökhan Aksan, Oguz Kağan Unal, Rukiye Nar, Korhan Soylu

**Affiliations:** Department of Cardiology, State hospital Aksaray, Aksaray, Turkey; Department of Cardiology, State hospital Gazi, Samsun, Turkey; Department of Endocrinology, State hospital Aksaray, Aksaray, Turkey; Department of Biochemistry, State hospital Aksaray, Aksaray, Turkey; Department of Cardiology, Scholl of Medicine, 19 Mayıs University, Samsun, Turkey

**Keywords:** Atherosclerosis, Diabetes mellitus, Transthoracic echocardiography

## Abstract

**Aim:**

Gestational diabetes mellitus (GDM) is associated with cardiovascular diseases; however, the relationship between epicardial fat thickness (EFT) and GDM remains unclear. The present study evaluates and compares EFT using transthoracic echocardiography in pregnant women with GDM.

**Materials and methods:**

This cross-sectional study included 129 pregnant women in the third trimester: 65 with GDM (GDM group) and 64 with uncomplicated pregnancies (control group). As defined by the World Health Organization, the diagnosis of GDM was based on an abnormal 2-h oral glucose tolerance test (OGTT) results. We used echocardiography to measure EFT in blood samples for all the participants.

**Results:**

The postprandial blood glucose level was significantly higher in the GDM group than in the control group (P < 0.001). There were no significant differences in BMI, heart rate, systolic and diastolic blood pressure or lipid parameters between the groups. In the GDM group, isovolumic relaxation time (IVRT) parameters were significantly higher than in the control group. EFT was significantly higher in the GDM group (P < 0.001) and was correlated with postprandial glucose, BMI, age, and heart rate in both the groups. Only postprandial glucose and BMI remained significantly associated with EFT after multiple stepwise regression analysis.

**Conclusion:**

Echocardiographically measured EFT was significantly higher in the patients with GDM. The findings show that EFT was strongly correlated with postprandial glucose.

## Introduction

Gestational diabetes mellitus (GDM) is impairment in carbohydrate tolerance that begins or is recognized for the first time during pregnancy [1]. It has an incidence of 1-14% [2, 3]. GDM is not just a pregnancy disorder, but patients with GDM are also at risk of developing type 2 diabetes mellitus (DM) postpartum [4]. The relationship between type 2 DM and cardiovascular diseases is well-known [5–8]. Since, the preclinical markers of the atherosclerotic process are observed just before the development of type 2 DM, cardiovascular risk in females can be complicated by GDM.

Epicardial fat tissue is an active tissue that originates from the same embryogenic layer as visceral adipose tissue and contributes to the energy supply of the heart and its surrounding tissues, and secretes hormones adiponectin and leptin. Epicardial fat thickness (EFT) is associated with the thickness of visceral fat tissue [9]. In addition to being an anatomical barrier, epicardial fat tissue is also a local reserve for proinflammatory cytokines [10] and is related to obesity, hypertension, insulin resistance and coronary artery disease [11–16]. Currently, EFT measurement is performed via transthoracic echocardiography (TTE), magnetic resonance imaging (MRI) and multi-slice computed tomography (CT) [17]. Measurement of EFT via TTE is quite easy and is used for cardiovascular risk assessment in routine practice [18].

The increase in EFT is associated with an increase in the incidence of insulin resistance and DM [11]; however, the relationship between GDM and EFT has not yet been evaluated. Since, GDM is associated with a decrease in insulin sensitivity or increase in insulin resistance, EFT measurement during the pregnancy can be a significant determinant of GDM. The present study aimed to evaluate and compare EFT using transthoracic echocardiography in pregnant women with and without GDM.

## Materials and methods

The subjects were selected from pregnant women referred to our clinics between January 2013 and August 2013 for routine pregnancy monitoring and were diagnosed with GDM. OGTT was conducted in patients with blood glucose levels over 140 mg/dL during 24–28 gestational weeks. OGTT was measured one hour after the patients were given water with 50 g glucose. Gestational diabetes was diagnosed if any two of the following values were found: fasting blood glucose levels ≥92 mg/dL, blood glucose levels ≥180 mg/dL in the first hour and ≥153 mg/dL during the second hour [19].

This study was approved by the local ethical committee. All subjects who participated in the study were informed about the study procedures and they signed an informed consent. We excluded women with a history of any previous glucose impairment (type 1 diabetes, type 2 diabetes, or gestational diabetes), previous or current blood pressure disorders and heart arrhythmia. Moreover, subjects with a history of the use of corticosteroids in the previous three months, tocolytic drugs, and serious maternal illnesses such as cancer, endocrinological disorders, chronic inflammation (systemic lupus erythematosus and rheumatoid arthritis), and those who were carrying more than one fetus, or had a liver or renal disease were also excluded.

Detailed physical examination was performed, blood pressure measurements were obtained and body mass index (BMI) was calculated in all subjects. Echocardiographic analyses were performed and blood samples were obtained from all the subjects.

### Echocardiographic investigation

All of the participants underwent TTE using a VIVID 3 (General Electric) device. The patients were evaluated while in the left lateral decubitus position following a five-minute rest. As recommended by the American Society of Echocardiography [20], left ventricle end-diastolic and end-systolic diameters, and the left ventricle ejection fraction (LVEF) were measured via M-mode echocardiography using standard windows and the parasternal long-axis view. Left ventricle systolic functions, left ventricle wall movements, and mitral, aortic, tricuspid, and pulmonary valve structures and insufficiency were evaluated with a 2-dimensional color Doppler ultrasonography. While examining diastolic functions, transmitral flow samples were written at a rate of 100 mm s^-1^ by placing the Pulse Wave (PW) Doppler cursor 1 cm above the mitral annular line in apical four-chamber sections.

EFT was defined as the echo-free space between the outer wall of the myocardium and the visceral layer of the pericardium. EFT was measured perpendicularly from the free wall of the right ventricle at end-diastole in three cardiac cycles, as previously described [21, 22]. The maximum EFT was measured from the point on the free wall of the right ventricle along the midline of the ultrasound beam perpendicular to the aortic annulus. For mid-ventricular parasternal short-axis assessment, maximum EFT was measured from the free wall of the right ventricle along the midline of the ultrasound beam, perpendicular to the intraventricular septum at the mid-choral level and the tip of the papillary muscles as the anatomic landmark. The intraobserver variability coefficient was calculated as 0.95.

### Blood collection and measurement

Blood samples from All the participants were obtained between 0800 and 1000 following 8–12 h of fasting. We used 8-mL vacuum tubes with gel to collect blood samples from antecubital veins. The samples were then centrifuged at 4000 × *g* for 10 min to separate the serum. Biochemical parameters were measured using an Abbott ARCHITECT c8000 (Abbott Laboratories, USA) auto analyzer and commercial kits. Hematological parameters were examined using an Abbott CellDyn 3700 (Abbott Laboratories, USA) device via the laser and impedance method.

### Statistical analysis

Statistical analyses were carried out using the Statistical package for Social Sciences for Windows version 15.0 (SPSS, Chicago, IL, USA). Descriptive statistics for each variable were determined. Results for continuous variables were demonstrated as mean ± standard deviation. Statistically significant differences between groups were determined by the chi-square test for categorical variables and unpaired Student’s t-test for continuous variables. Associations between the variables were explored using the Pearson correlation and Spearman’s rho (for data that were not normally distributed). A multiple stepwise line regression analysis was used to determine the contribution of various factors to EFT. Univariate analysis was performed and variables with p < 0.10 were entered into a backward stepwise multivariate logistic regression analysis to assess independent predictor for the presence of GDM. A *p*-value less than 0.05 was considered significant.

## Results

The study included 129 pregnant women: 65 in the GDM group and 64 in the control group. Mean age in the GDM group was 29.7 ± 5.4 years versus 30.3 ± 4.4 years in the control group (*p* = 0.65). Heart rate, BMI and systolic and diastolic blood pressure measurements were not significantly different between the GDM and control groups. Fasting and postprandial blood glucose levels were significantly higher in the GDM group than in the control group (*p* <0.001); however, there were not any significant differences in lipid parameters between the groups (Table 1).

**Table 1:** **Baseline clinical and laboratory characteristics of study population and comparison between the groups**

Variables	GDM group (n: 65)	Control group (n: 64)	***p***-value
Age (year)	29.7 ± 5.4	30.3 ± 4.4	0.651
Body mass index (kg/m^2^)	27.4 ± 3.4	26.3 ± 3.4	0.082
Heart rate (beats/minute)	89.3 ± 11.5	88. 9 ± 7.3	0.695
Mean systolic blood pressure (mmHg)	112.3 ± 12.8	112.6 ± 13.1	0.551
Mean diastolic blood pressure (mmHg)	71.0 ± 9.7	69.7 ± 11.1	0.991
Serum glucose (mg/dL)	98.8 ± 14.8	89.1 ± 9.3	<0.001
Postprandial serum glucose (mg/dL)	192.4 ± 37.4	132.2 ± 8.7	<0.001
Triglyceride (mg/dL)	184 ± 116	168 ± 58.5	0.411
Low-density lipoprotein cholesterol (mg/dL)	103.3 ± 36.7	105.3 ± 35.7	0.574
High-density lipoprotein cholesterol (mg/dL)	35.9 ± 7.6	36.2 ± 8.7	0.934
Total cholesterol (mg/dL)	179.2 ± 40.6	177.4 ± 37.9	0.891

Abbreviations: *GDM* Gestational diabetes mellitus.

Interventricular septum thickness (IVS), posterior wall thickness (PW), left ventricular end-systolic diameter (LVESD), left ventricle end-diastolic diameter (LVEDD), LA diameter and LVEF (*p* > 0.05) were not significantly different between the GDM and control groups. IVRT was significantly extended in the GDM group compared to the control group (80.8 ± 26.4 vs. 71.59 ± 17.5; *p* = 0.020); however, E, A, and E/A rate, E/E’ rate and deceleration times were significantly different (Table 2).

**Table 2:** **Comparison of echocardiographic parameters between the groups**

Variables	GDM group (n: 65)	Control group (n: 64)	***p***-value
Left ventricle end-diastolic diameter (mm)	46.0 ± 5.1	46.6 ± 4.5	0.675
Left ventricle end-systolic diameter (mm)	28.8 ± 6.6	30.4 ± 3.0	0.224
Left ventricular ejection fraction (%)	64.6 ± 5.1	62.9 ± 4.5	0.205
Interventricular septum (mm)	8.8 ± 1.4	8.7 ± 1.6	0.682
Posterior wall (mm)	8.9 ± 1.2	9.0 ± 1.5	0.823
Right ventricular diameter (mm)	27.4 ± 2.6	27.5 ± 2.4	0.654
Left atrium diameter (mm)	32.9 ± 3.8	32.8 ± 2.7	0.784
E wave	0.75 ± 0.2	0.77 ± 0.2	0.685
A wave	0.59 ± 0.1	0.57 ± 0.1	0.065
Deceleration time	192.6 ± 47.3	181.4 ± 25.6	0.112
isovolumetric relaxation time	80.8 ± 26.4	71.59 ± 17.5	0.020
E/A	1.29 ± 0.4	1.38 ± 0.3	0.141
E/E’	6.7 ± 1.8	6.1 ± 2.3	0.193
Epicardial fat thickness (mm)	7.2 ± 2.5	5.6 ± 1.7	<0.001

Simple logistic regression analysis revealed that post-prandial glucose (mg/dL) (OR = 1.566, 95% CI: 0.815-3.236; *p* < 0.001) and epicardial fat thickness (mm) (OR = 1.624, 95% CI: 0.815-3.236; *p* < 0.001 showed a trend (*p* < 0.10) toward an association with the presence of GDM (Table 3). We entered these variables into a backward stepwise multivariate logistic regression model, which demonstrated that post-prandial glucose and epicardial fat thickness were significant and independent predictors of GDM(OR = 1.625, 95% CI: 0.988-2.786; p < 0.001 and OR = 1.524, 95% CI: 0. 0.715-2.958; *p* < 0.001) (Table 3).

**Table 3:** **Logistic regression analysis for the presence of GDM in pregnancy**

	Simple regression OR (95% CI)	***p***-value	Multiple regression OR (95% CI)	***p***-value
Age (years)	0.979 (0.915-1.048	0.538		
Post-prandial glucose (mg/dL)	1.566 (0.965-2.862)	<0.001	1.625 (0.988-2.786)	<0.001
Body mass index (kg/m^2^)	1.125 (0.848-1.494)	0.414		
Heart rate (beats/minute)	1.095 (1.011-1.186)	0.266		
Epicardial fat thickness (mm)	1.624 (0.815-3.236)	<0.001	1.524 (0.715-2.958)	<0.001

**Figure 1:**
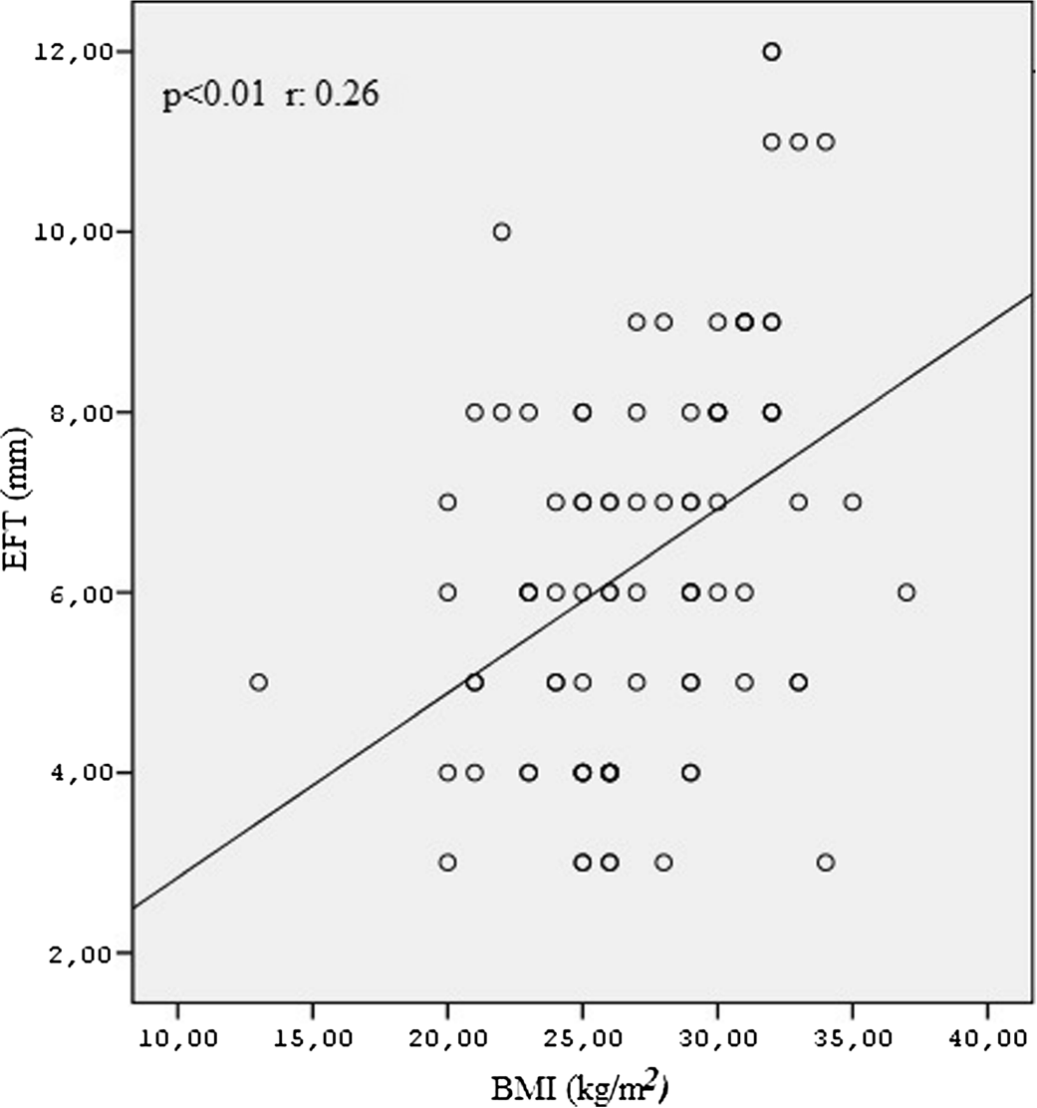
**The correlation analysis of postprandial glucose levels and epicardial fat thickness in both the groups.**

**Figure 2:**
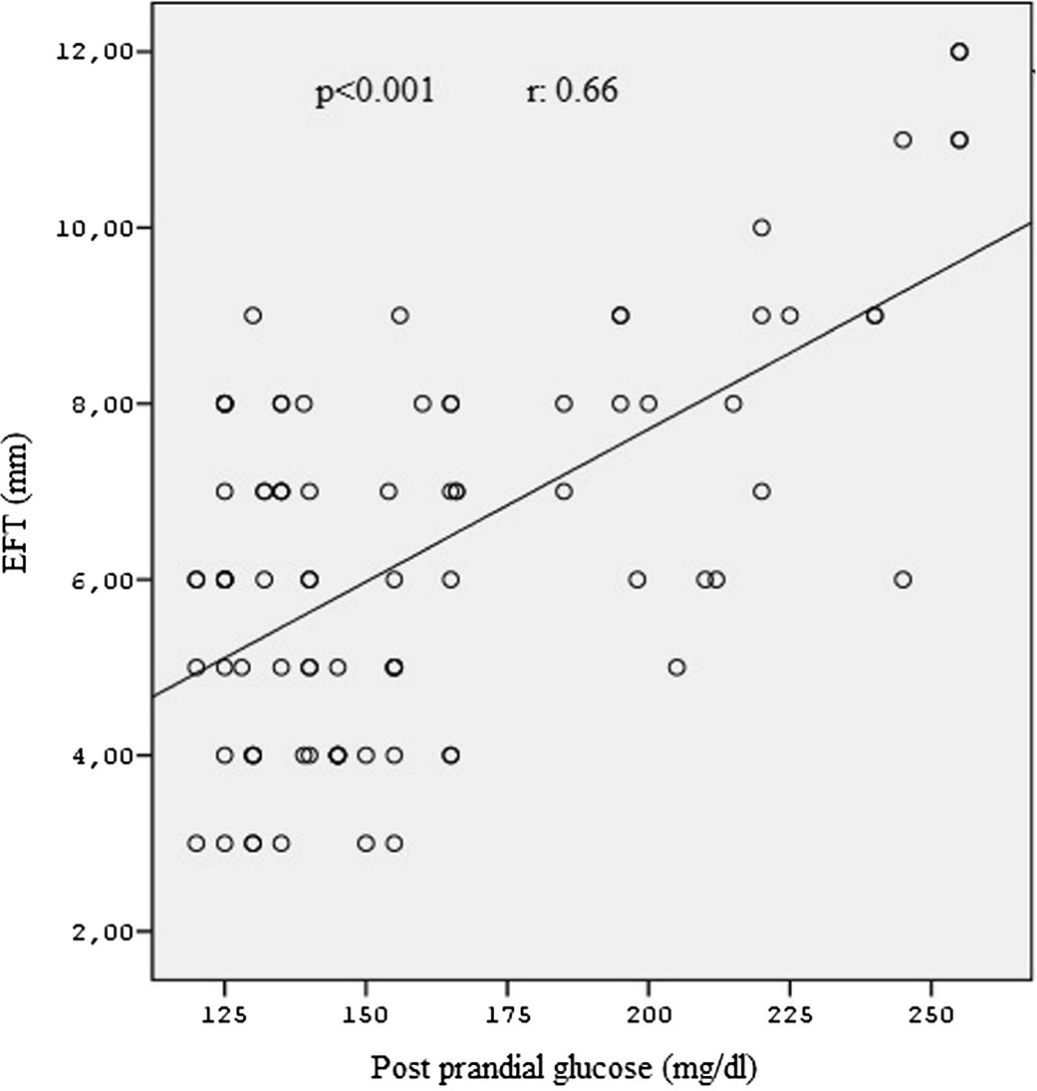
**The correlation analysis of BMI levels and epicardial fat thickness in both the groups.**

Pearson’s correlation analysis showed that EFT was positively correlated with the post-prandial glucose level (*p* < 0.001) and BMI in both the groups (*p* < 0.01) (Figures 1 and 2). Correlation between EFT and other variables are shown in Table 4. Multiple stepwise regression analysis showed that only post prandial glucose (r = 0.627, *p* < 0.001) and BMI (r = 0.264, *p* < 0.01) significantly associated with serum EFT.

**Table 4:** **The univariate correlations of the epicardial fat thickness**

Variables	r-value	***p***-value
Postprandial glucose (mg/dL)	0.66	<0.001
Body mass index (kg/m^2^)	0.26	<0.01
Heart rate (beats/minute)	0.25	0.01
Age (years)	0.19	0.04

Abbreviations: *BMI* Body mass index.

## Discussion

We used TTE to measure EFT in pregnant women with and without GDM and observed two significant findings: EFT was significantly higher in the GDM group, and it was significantly correlated with the post-prandial glucose level.

Among the factors associated with GDM, visceral obesity is a major risk factor. Progressive increase in intra-abdominal fat tissue causes insulin resistance in liver and adipose tissue, and, as a result, causes glucose intolerance, low-level HDL, elevated triglycerides and hypertension [23, 24]. Since, epicardial fat tissue originates from visceral fat tissue, the present study was conducted to investigate the hypothesis that EFT might be elevated in patients with GDM. Several methods are used to measure EFT in systole and diastole. Some researchers favor investigator-dependent methods such as echocardiography, whereas others prefer investigator-independent methods such as multislice computed tomography. In the present study, we used TTE to measure EFT.

Epicardial adipose tissue is not only a physiological barrier, but is also a bioactive organ that produces several inflammatory cytokines and adipokines [25, 26]. These inflammatory cytokines trigger physiopathological mechanisms such as insulin resistance, endothelial dysfunction, atherosclerosis, and obesity. There is a relationship between EFT, abdominal visceral obesity, subclinical atherosclerosis, impaired fasting glucose, type 1 DM, and hypertension [27–31]. The negative glucose profile in epicardial fat tissue is affected by the levels of glucose transporter-4 and retinol-binding protein [32]. Moreover, mRNA expression is related to epicardial fat, plasma insulin, and insulin-resistance-related molecule resistance [25]. The negative effects of EAT on glucose metabolism are also associated with the studies performed in patients with DM [11].

Caliskan et al. [33] showed that patients who previously had GDM showed elevated EAT compared to patients in the control group. Moreover, the authors concluded that patients with a history of GDM also had atherosclerosis.

Elevated EFT can further complicate GDM because EFT is also a risk factor for coronary artery disease. Kim et al. [34] used cardiovascular magnetic resonance (CMR) to show that an increase in EFT is an independent risk factor for coronary artery stenosis in patients with asymptomatic type 2 DM. Wang et al. [35] used cardiac multislice CT to describe the relationship between EFT volume and metabolic syndrome and coronary atherosclerosis. In another study, Bachar et al. [36] used a multidetector CT to investigate EFT, coronary calcium score and coronary artery stenosis, and reported that EFT is a strong predictor of coronary artery disease.

DM affects the diastolic functions of the heart, and uncontrolled hyperglycemia provokes the diastolic left ventricular dysfunction in type 2 diabetic patients without cardiac involvement [37, 38]. Left ventricular diastolic function changes are associated with fasting plasma glucose levels and glycohemoglobin concentrations under the diabetes threshold [39]. The IVRT elongation observed in the GDM group in the present study can be attributed to the negative effects of hyperglycemia on the diastolic functions of the heart.

In the present study, we observed a strong positive correlation between EFT and postprandial glucose levels. Echocardiography and CT studies show a strong correlation between fasting plasma glucose and EFT [40]. In the present study, we showed a relationship between the postprandial glucose level and EFT, which might have been due to the relationship between EFT and hyperglycemia-insulin resistance, as described above. Our study corroborated the results of a previous study that showed that BMI and EFT have a weak correlation. Bettencourt et al. [41] showed that EFT is directly related to gender, age, BMI, waist circumference, abdominal visceral fat tissue, and atherosclerotic load. EFT is associated with BMI in females with DM [11] and was higher in females with a BMI >27 kg m^-2^ [16]. Moreover, EFT is an independent predictor of intra-abdominal visceral fat tissue [42–44]. The correlation between EFT and BMI is an expected finding, as EFT is a parameter indicative of adipose tissue. However, our study showed that BMI is weakly correlated with EFT. This can be attributed to the weight gain and the changes in body composition during pregnancy. These changes include the changes in adipose tissue and RBC mass, body water, uterine and breast tissue, and the products of conception including the fetus, placenta and amniotic fluid [45]. The increased visceral adipose tissue has a decreased effect on weight gain during pregnancy, which may cause the weak correlation between BMI and epicardial adipose tissue.

### Limitations of the study

Measurement of EFT is routinely used during echocardiographic examination. This is an observational study. In addition to the adipose tissue, pregnancy-associated weight gain and significant changes in body composition affect EFT related parameters such as waist circumference, anthropometric measurements and BMI. Studies involving pre- and post-pregnancy follow-ups may help us understand the cause and effect relationship.

## Conclusion

This study shows that TTE measured EFT is higher in patients with GDM. Moreover, a strong correlation between EFT and the postprandial glucose levels was found. These findings can be helpful to understand the physiopathology of GDM; however, further studies are necessary to support this conclusion.
